# An investigation into the potent anticancer, antimicrobial, and anti-inflammatory activities of a *Punica granatum* nanoemulgel

**DOI:** 10.37796/2211-8039.1663

**Published:** 2025-09-01

**Authors:** Ahmad M. Eid, Murad Abualhasan, Yara Khaliliya, Zeina Sinan, Aya Khaliliya

**Affiliations:** Department of Pharmaceutical Chemistry and Technology, Faculty of Pharmacy, An-Najah National University, Nablus, Palestine

**Keywords:** Anticancer, Antimicrobial, Nanoemulgel, Self-emulsifying system, *Punica granatum*

## Abstract

**Purpose:**

The objective of this study is to formulate a nanoemulgel using *Punica granatum* (*P. granatum*) seed oil and study the antibacterial, anticancer, and anti-inflammatory properties.

**Methods:**

The process to formulate a nanoemulsion from *P. granatum* seed oil involved using the self-nanoemulsifying technique, with Span 80 and Tween 80 serving as the emulsifying agents. Carbopol hydrogel was combined with the nanoemulsion to produce the nanoemulgel. The particle size, polydispersity index (PDI), rheological behavior, antibacterial, cytotoxic, and anti-inflammatory properties were subsequently examined.

**Results:**

The nanoemulsion formulation with a PDI of 0.229 and a particle size of 189.44 nm was identified as the optimal formulation. The *P. granatum* seed oil nanoemulgel showed significant effects on MRSA, *K. pneumoniae*, and *C. albicans*, with zone inhibition diameters of 29 ± 1.1 mm, 26 ± 1.8 mm, and 18 ± 0.7 mm, respectively, and significant activity against LX-2, B16-F1, Hep-3B, and HeLa cancer cell lines with IC_50_ values of 169.82 ± 2.7, 39.81 ± 0.8, 61.65 ± 1.2, and 25.11 ± 1.3 μg/mL, respectively, which were superior to those of the original oil. Regarding its anti-inflammatory effects, *P. granatum* seed oil demonstrated activity against both COX-1 and COX-2, with greater selectivity for COX-1.

**Conclusions:**

Consequently, a novel *P. granatum* seed oil nanoemulgel was developed, representing a promising step forward in the development of pharmacological dosage forms.

## 1. Introduction

In recent times, scientists have begun to employ herbalism, which is sometimes referred to as folk medicine, which is a traditional practice involving the use of botanicals and plant extracts to treat a variety of diseases, including inflammation, mental disorders, prostate issues, cardiovascular disease, and immune system support. Furthermore, an extensive proportion of medical professionals advocate for the application of botanical remedies, herbal treatments, and complementary and alternative medicine (CAM) methodologies in the management of specific diseases. Various botanical components have been used in herbal therapy, including fruits, seeds, berries, roots, foliage, bark, and blossoms, and occasionally the entire plant [1]. The initial application of medicinal plants followed an instinctual pattern, similar to how animals behave [2]. At present, botanicals find application in the treatment of acute and chronic diseases, covering a wide variety of problems and concerns such as prostate concerns, cardiovascular disease, depression, inflammation, and immune system enhancement [3]. Presently, numerous “conventional” medical professionals support the efficacy of complementary and alternative medicine (CAM) therapies, botanicals, and herbal products to treat specific diseases [4].

Pomegranate, scientifically called *Punica granatum* (*P. granatum*) of the *Punicaceae* family, is one of the most widely used medicinal plants globally, widely used to treat various diseases. The significant bioactive compounds in *P. granatum* seed oil, such as punicalagins, ellagic acid, punicic acid, and other polyphenols, have generated attention for their potential use in treating inflammation and cancer. Pomegranate oil exhibits a variety of anti-cancer properties. Punicalagins and ellagic acid have strong antioxidant activity by scavenging free radicals and reducing oxidative stress, protecting cells from DNA damage that is essential for the development of cancer. Furthermore, its anti-inflammatory properties prevent chronic inflammation, which is essential for the growth of cancer, and inhibit activities including angiogenesis, metastasis, and cell division. The oil promotes cell death through apoptosis induction, which is made possible by compounds like punicic acid. This process is essential for eliminating abnormal cells that could support the growth of cancer. Its immune-modulating powers also improve immune surveillance and activate important immune cells, which enhances the detection and destruction of malignant cells [1,5,6].

*P. granatum* seed oil demonstrates potent antiinflammatory properties through several mechanisms. It inhibits cyclooxygenase (COX) and lipoxygenase (LOX) enzymes, which play crucial roles in the production of pro-inflammatory mediators like prostaglandins and leukotrienes, thereby mitigating inflammation and associated symptoms. Additionally, compounds in pomegranate oil suppress the activation of NF-κB, a key regulator of inflammation and immune responses, reducing the expression of pro-inflammatory genes and cytokines. Its capacity to modulate cytokine production and activity further helps regulate the inflammatory cascade and maintain immune homeostasis [7,8].

Overall, the multifaceted anti-cancer and antiinflammatory attributes of pomegranate oil position it as a promising adjunct therapy in cancer treatment and inflammation management. Nevertheless, further research, including clinical trials, is needed to determine its effectiveness, optimal dosage, and potential interactions with conventional treatments. It is essential to consult healthcare professionals before integrating pomegranate oil into cancer or inflammation treatment protocols [7,8].

Nanotechnology and nanoemulgel formulations provide a transformative approach to improving the effectiveness and stability of herbal medicines. By reducing herbal particle size to the nanometer scale, these technologies increase surface area, improving the solubility and bioavailability of active constituents. Encapsulation within nanoemulsion droplets enables targeted delivery to specific tissues or cells, optimizing herbal compound absorption. Additionally, nanoemulgel matrices protect against degradation factors, extending shelf life and preserving potency. Enhanced skin penetration facilitates transdermal delivery, while controlled release profiles ensure sustained therapeutic effects. Additionally, these formulations allow for synergistic combinations of herbal compounds or integration with conventional drugs, potentially augmenting therapeutic outcomes. Overall, the utilization of nanotechnology and nanoemulgel formulations represents a promising frontier in herbal medicine, offering advancements in efficacy and versatility for improved healthcare solutions [9].

This study aims to develop a nanoemulgel formulation incorporating *Punica granatum* (pomegranate) seed oil and evaluate its multifaceted biological activities, including antibacterial, antiinflammatory, and anticancer effects. The goal is to optimize the formulation for enhanced bioavailability and efficacy, providing a potential therapeutic platform for treating bacterial infections, inflammatory conditions, and cancerous growths.

## 2. Materials and methods

### 2.1. Materials

Mueller-Hinton agar, produced by Becton Dickinson and Company in Le Pont-de-Claix, France, was used in addition to *P. granatum* seed oil extracted from seeds harvested in various Palestinian agricultural regions. Dimethyl sulfoxide (DMSO) was obtained from Riedel-de Haën, Germany, and carboxyvinyl polymer (Carbopol 940) was supplied by CBC Co., Ltd., Tokyo, Japan. The *P. granatum* seeds were examined at An-Najah National University's Pharmacy Department in the Faculty of Medical and Health Sciences by Dr. Nidal Jaradat. The results were recorded and assigned the voucher specimen code Pharm-PCT-2721 for future reference.

The plant material was extracted using HPLC-grade solvents. Pig pancreatic amylase was supplied by MP Biochemicals (Illkirch, France), while acarbose, orlistat, starch, PNPP (pnitrophenyl palmitate), and porcine pancreatic lipase were supplied by Sigma-Aldrich (USA).

All materials used in the experiments were of analytical quality.

### 2.2. *P. granatum* preparation and seed oil extraction

Proper authorization was obtained to gather the plant material. The laboratory personnel from An-Najah National University's Pharmacy Department gathered and identified the botanical specimen. To extract the seeds, we collected *P. granatum* fruits from various locations throughout the West Bank, Palestine. Following a previously established scientific process [10], the seeds were ground into a fine powder and dried in the shade for about 20 days at 25 ± 3 °C and 55 ± 4 % relative humidity (RH). Next, we weighed 50 g of the ground seeds and soaked them in a mixture of 250 mL of pure hexane (a nonpolar organic solvent) and 250 mL of 70 % ethanol solution (a polar solvent). The soaked seeds were kept at room temperature in a shaking bath for 24 h. The extract was then separated into two layers using a separatory funnel. The solvent in the organic layer was subsequently removed under air pressure to concentrate the extract.

#### 2.2.1. Preparation of *P. granatum* seed oil nanoemulsion

The optimum nanoemulsion formulation was optimized by screening a variety of selfnanoemulsifying formulations with varying proportions of co-surfactant (Span 80), surfactant (Tween 80 and Tween 20), and *P. granatum* seed oil. This process led to the construction of two ternary-phase diagrams. Tween 80, Span 80, and oil composed the first diagram, while Tween 20, Span 80, and oil composed the second. For each formulation, we weighed the three ingredients and used a vortex mixer (VELP Scientifica, Europe) to mix them for 2 min. The formulation was self-emulsified by gently mixing it in 50 mL of distilled water and subsequently analyzed for droplet size and polydispersity index (PDI) using a particle size analyzer (Brookhaven Instruments, NanoBrook Omni, New York, USA) [11].

#### 2.2.2. Droplet size and polydispersity index analysis of *P. granatum* seed oil nanoemulsion

The *P. granatum* seed oil nanoemulsion droplet size and PDI were determined using a particle size analyzer (Brookhaven Instruments, NanoBrook Omni, New York, USA). Prior to measurement, we emulsified the formulation in distilled water [12]. We then selected the optimal nanoemulsion formulation based on the lowest PDI, the smallest droplet size, and the minimum amount of *P. granatum* seed oil used. Every measurement was conducted at room temperature in triplicate.

#### 2.2.3. Preparation of Carbopol 940 hydrogel

Carbopol 940 was weighed and added to distilled water at a 4 % concentration to produce the hydrogel, and the mixture was continuously stirred using a lab mixer until it became homogeneous and smooth. To ensure that the gelation process was completed. The mixture was left for 24 h to complete the gelation process, and the pH of the hydrogel was adjusted to 6 using sodium hydroxide.

#### 2.2.4. Formulation of *P. granatum* seed oil nanoemulgel

The *P. granatum* seed oil nanoemulgel formulations were prepared by incorporating varying concentrations (0.4 %, 0.6 %, 0.8 %, and 1 %) of the Carbopol hydrogel into *P. granatum* seed oil nanoemulsion. The formulations were thoroughly mixed until they became homogenous. Next, a master size analyzer (Brookhaven Instruments, Nano Brook Omni, New York, NY, USA) was used to measure the droplet size, polydispersity, and zeta potential.

#### 2.2.5. Physical characterization of *P. granatum* seed oil nanoemulgel

Several physical properties of the *P. granatum* seed oil nanoemulgel were examined visually during the preparation process. These properties included homogeneity, phase separation, visual appearance, distribution, and consistency. The pH values were measured using a pH meter (CG 820, Schott Gerate GmbH, Hofheim, Germany).

#### 2.2.6. Analysis of *P. granatum* seed oil nanoemulgel zeta potential

Using the zeta potential approach, we measure the surface charge and particle dispersion stability of the *P. granatum* seed oil nanoemulgel using the NanoBrook Omni. The zeta potential was measured three times for each concentration of Carbopol, and the average values were plotted against the corresponding concentrations [13].

#### 2.2.7. Rheological measurement of *P. granatum* seed oil nano-emulgel

The *P. granatum* seed oil nanoemulgel formulations were produced with thickening agents in different concentrations (0.4, 0.6, 0.8, and 1 %) of Carbopol 940. A viscometer (Brookfield DVI, Middleboro, MA, USA) was used to measure the rheological behavior of the *P. granatum* seed oil nanoemulgel formulations. It operated with a shear rate range of 0–100 rpm and utilized a 7-size spindle. The measurements were carried out in triplicate at room temperature [12].

### 2.3. Antimicrobial evaluation of *P. granatum* seed oil and its nanoemulgel

#### 2.3.1. Culture media

As the culture medium, Mueller Hinton agar was produced in France by Becton, Dickinson, and Sparks Co., Sparks, MD, USA. Each liter of sterile water contained the following components: 17.5 g of casein acid hydrolysate, 1.5 g of starch, 2 g of beef extract, and 17 g of agar. Once the components had been thoroughly combined, they were brought to a simmer with gentle stirring to facilitate dissolution. Following a 20-min autoclaving at 121 °C, the agar was allowed to cool before being transferred to new Petri plates. In order to maintain uniformity in height and breadth, a flat surface was employed. Agar was finally preserved at 4–8 °C. For the assessment of antimicrobial and antifungal properties, agar diffusion was employed.

Four apertures, each with a diameter of 6 mm, were punched into the agar plates; these holes were designated A, B, C, and D. In hole B, *P. granatum* seed oil was introduced, whereas in hole A, only DMSO was utilized. Hole C was filled with *P. granatum* seed oil nanoemulgel, whereas hole D was filled with an emulgel devoid of oil. For the antibacterial assessment, the plates were incubated at 37 °C for 24 h, while for the antifungal evaluation, they were at 25 °C. The diameter of the inhibitory zone was measured so that its antibacterial and antifungal properties could be determined [14]. DMSO serves as a negative control, and the emulgel without oil helps assess the effect of the oil in the formulation.

#### 2.3.2. Antibacterial and antifungal

Six different bacteria MRSA, *Proteus vulgaris*, *Escherichia coli*, *Klebsiella pneumoniae*, *Pseudomonas aeruginosa*, and *Staphylococcus aureus* were used in the antibacterial test utilizing the microdilution method for *P. granatum* oil and nanoemulgel compared with ampicillin antibiotics. All of these bacteria are available in the ATCC. Moreover, *Candida albicans* was utilized appropriately for the antifungal examination and compared to fluconazole.

### 2.4. Cytotoxicity evaluation of *P. granatum* seed oil and its nanoemulgel

The cytotoxic potential activities of *P. granatum* seed oil and its nanoemulgel have been evaluated on several cancer cell lines, including human B16-F1 melanoma cells, human LX-2 and Hep-3B hepatocellular carcinoma cells, and HeLa human cervical epithelioid carcinoma cells. The cells were cultured in RPMI 1640 medium (Biological Industries, Cromwell, CT, USA), which were supplemented with 10 % fetal bovine serum, 1 % penicillin/streptomycin, and 1 % l-glutamine. Cell cultures were incubated at a temperature of 37 °C in a humid atmosphere with 5 % CO_2_ [15].

For the experimental configuration, cells were inoculated in triplicate into 96-well plates utilizing 100 μL of each growth medium. The wells were filled with approximately 5000 cells. Following a 24-h incubation period, fresh medium containing *P. granatum* seed oil was introduced into the cultures at the following concentrations: 62.5, 125, 250, 500, and 1000 μg/mL. The cells were subsequently cultured for an additional 72 h. To assess potential anti-proliferative effects, the Cell Titer 96® Aqueous One Solution Cell Proliferation (MTS) Assay (Promega Corporation, Madison, WI, USA) was utilized in adherence to the guidelines provided by the manufacturer.

After treatment, MTS solution was added to each well and incubated at 37 °C for 2 h. Absorbance was measured at 490 nm to evaluate the plant oil's effect on cell proliferation [16].

### 2.5. COX enzyme evaluation of *P. granatum* seed oil and its nanoemulgel

In order to investigate whether *P. granatum* seed oil might inhibit the conversion of arachidonic acid (AA) to prostaglandin H_2_ (PGH_2_), we utilized a Cayman Chemical Company (USA) COX inhibitor screening assay reagent (Item No. 460104). The 50 % inhibitory concentration (IC_50_) was determined by examining two concentrations (50 and 300 μg/mL) of each compound (*P. granatum* seed oil and its nanoemulgel) in duplicate experiments.

The degree of inhibition exhibited by the sample was assessed by employing the best-fitting line derived from a multiple regression analysis. This was achieved in accordance with the instructions in the assay kit manual by utilizing a standard curve comprising eight different concentrations of prostaglandins, in addition to a non-specific binding sample and a maximum binding sample. Inhibition percentages at each concentration were utilized to determine the IC_50_ [17].

## 3. Results

### 3.1. Analysis of *P. granatum* seed oil nanoemulsion droplet size and PDI

**Fig. 1:**
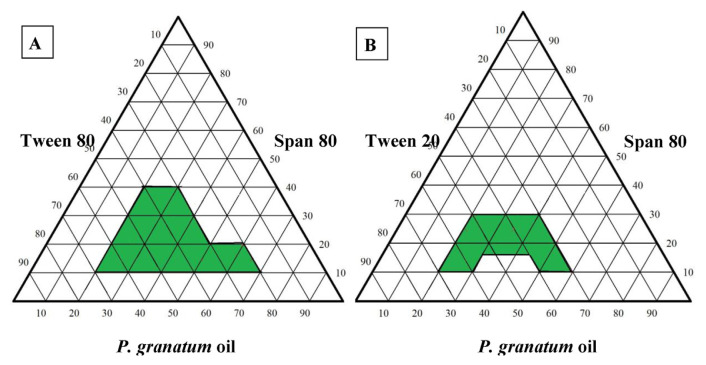
Pseudo ternary phase diagrams of *P. granatum* seed oil nanoemulsion, (A: T80, S80, and oil) and (B: T20, S80, and oil).

By using various concentrations for the surfactant (Tween 80), co-surfactant (Span 80), and *P. granatum* seed oil, the ternary phase diagrams were prepared to determine the suitable formulation of the selfnanoemulsifying system with a PDI <0.3 and a droplet size below 200 nm (Fig. 1). Many formulations were compared to each other; the selected optimum formulation was 50 % Tween, 15 % Span 80, and 35 % *P. granatum* seed oil, as shown in Table 1, which processed a PDI of 0.229 ± 0.09 and a droplet size of 189.44 ± 2.1 nm.

Different concentrations of Carbopol 940 (0.4 %, 0.6 %, 0.8 %, and 1 % w/w) were used to create a nanoemulgel that included *P. granatum* seed oil. The swelling properties of Carbopol led to its selection as the gelling agent. The nanoemulsion formulation utilized Tween 80 and Span 80, respectively, as the surfactant and co-surfactant. Following that, the nanoemulgel was formed by combining this formulation with Carbopol 940 hydrogel and distilled water. The nanoemulgel formulation's particle size, viscosity, and size distribution were evaluated.

### 3.2. Impact of different carbopol concentrations on droplet size and PDI of *P. granatum* seed oil nanoemulgel

**Fig. 2:**
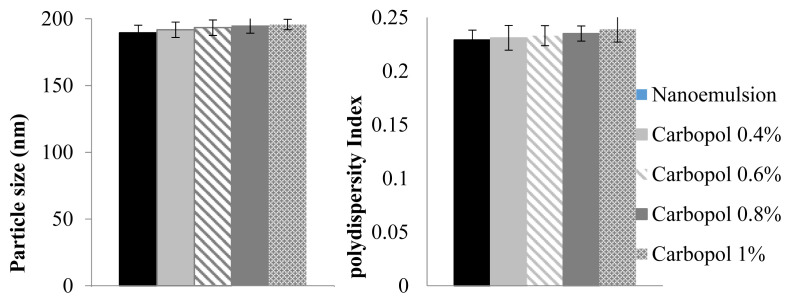
Droplet size and polydispersity index (PDI) of *P. granatum* seed oil nanoemulgel with different Carbopol concentrations.

According to the results in Fig. 2, the average particulate size was submicron, characterized by a narrow size distribution and a low PDI. In order to determine the most effective *P. granatum* seed oil nanoemulgel formulation with the smallest particle size and PDI, three distinct Carbopol 940 concentrations were employed in the nanoemulsion synthesis process: 0.4 %, 0.6 %, 0.8 %, and 1 %. The objective was to attain an optimal nanoemulsion by manipulating PDI and particle size.

### 3.3. Sensorial property analysis and physical characterization of *P. granatum* seed oil nanoemulgel

The nanoemulgel formulated with *P. granatum* seed oil should exhibit spreadability and ease of application. However, increasing the Carbopol concentration in the mixture resulted in decreased spreadability and increased difficulty in handling. Therefore, we selected 0.4 % Carbopol over 0.6 %, 0.8 %, and 1 % due to its lower concentration, which was less cumbersome. We observed minimal variation in spreadability among the different concentrations. The nanoemulgel maintained a pH of 6. The optimal formulation should possess a short emulsification time (less than 30 s) and high spreadability.

### 3.4. Zeta potential measurement of *P. granatum* seed oil nanoemulgel

**Fig. 3:**
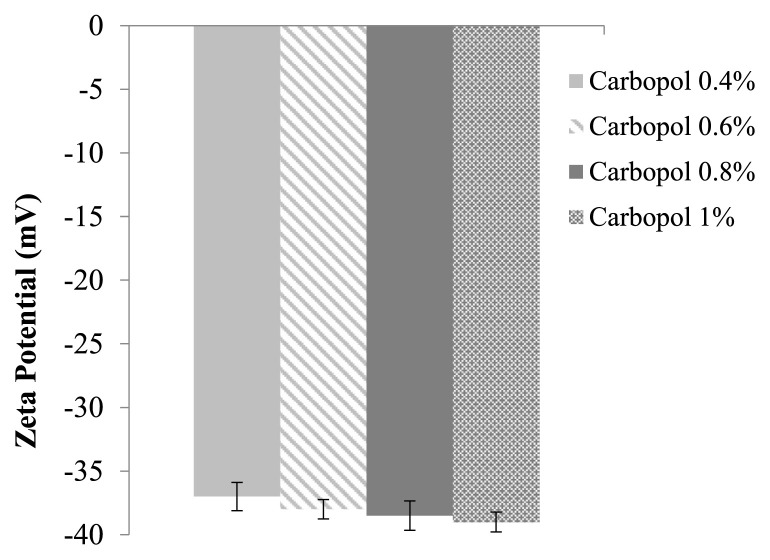
Zeta potential of *P. granatum* seed oil nanoemulgel formulations.

Fig. 3 illustrates that the zeta potential of each *P. granatum* seed oil nanoemulgel formulation was consistently below −40.

### 3.5. The rheological behavior of *P. granatum* seed oil nanoemulgel formulations

**Fig. 4:**
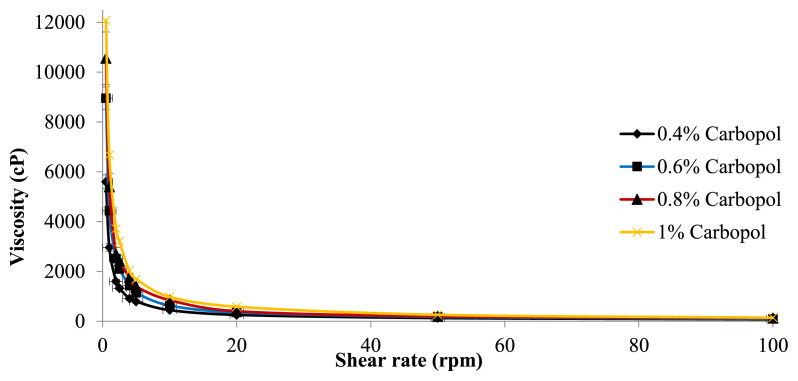
Rheological behavior of *P. granatum* seed oil nanoemulgel formulations.

The rheological characterization of semi-solid pharmaceutical preparations evaluates their functionality and quality by assessing their flow properties. A study on the rheological properties of nanoemulgel formulations containing *P. granatum* seed oil is illustrated in Fig. 4. The correlation between shear rate and viscosity decrease, as observed, indicates that the rheology of these compositions exhibits pseudoplastic behavior.

### 3.6. Antimicrobial activity of *P. granatum* seed oil and its nanoemulgel

The results of the antibacterial studies conducted on different gram-positive and gram-negative bacterial strains using *P. granatum* seed oil and its nanoemulgel were different from those observed when using control-positive antibiotics and antifungal agents like fluconazole and ampicillin. Based on the measurement of the zone of inhibition diameter (in millimeters), it was observed that the oil demonstrated a more pronounced effect on *K. pneumoniae* in comparison to the positive control drug (ampicillin), which had a diameter of 19 ± 1.5 mm. On the contrary, the nanoemulgel exhibited a more pronounced impact on MRSA and *K. pneumoniae* than both the oil and the positive control drug (ampicillin), as evidenced by the zone of inhibition diameters of 26 ± 1.8 mm and 29 ± 1.1 mm, respectively. Moreover, the impact of the oil on *Candida albicans* was comparable to that of the positive control drug (fluconazole), whereas the nanoemulgel demonstrated a more pronounced effect in comparison to the positive control drug (fluconazole), as evidenced by the 18 ± 0.7 mm diameter zone of inhibition in Table 2 below.

### 3.7. Anticancer activity of *P. granatum* seed oil and its nanoemulgel

In this investigation, we examined the potential anticancer properties of *P. granatum* seed oil and its nanoemulgel formulation using four distinct cancer cell lines: HeLa, Hep-3B, LX-2, and B16-F1. HeLa cells, originating from cervical cancer specimens, are recognized for their significant contribution to fatal cancer cases among women. Key factors associated with this cancer type include smoking, oral contraceptive use, and infection by human papillomavirus (HPV). Hep-3B cells, derived from a liver tumor of a 15-year-old Chinese male patient in 1975, are widely utilized in cancer research. Excessive alcohol consumption and smoking are prominent factors contributing to the development of this specific cancer type. B16-F1 cells, a murine melanoma cell line, are commonly employed in melanoma-related studies, given their origin from a melanoma tumor in a C57BL/6 mouse strain. Key factors contributing to melanoma include fair skin, light-colored hair, and eyes, presence of dysplastic nevi (atypical moles), exposure to certain chemicals, and genetic predisposition. LX-2 cells, a human hepatic stellate cell line, are frequently utilized in liver disease research, including studies on cancer and cirrhosis. These cells were derived from non-cancerous human hepatic tissue.

**Fig. 5:**
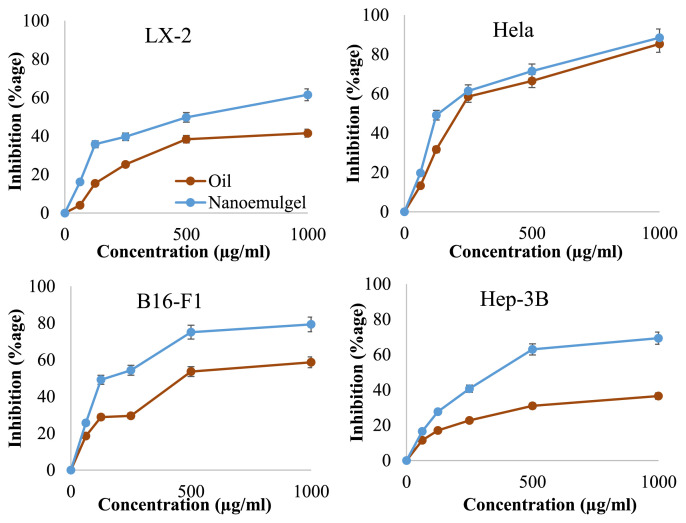
Cytotoxic effects of *P. granatum* seed oil and its nanoemulgel.

Following the cytotoxic tests, we obtained compelling findings, as delineated in Fig. 5. These findings elucidate the relationship between the concentration of *P. granatum* seed oil and its nanoemulgel, juxtaposed against the percentage of inhibited cancer cell growth. There was a discernible influence of both the oil and its nanoemulgel on these cancer cells, as evidenced by the escalated suppression of their growth with increasing concentrations of the oil and its nanoemulgel. This underscores the consequential impact of the oil and its nanoemulgel on the proliferation of these cancer cells.

**Fig. 6:**
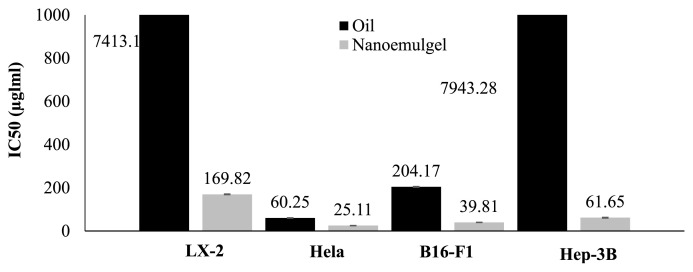
The IC_50_ values (μg/mL) of *P. granatum* seed oil and its nanoemulgel against different cancer cell lines.

Fig. 6 elucidates the IC_50_ values pertaining to *P. granatum* seed oil and its nanoemulgel concerning three distinct cancer cell lines. Their efficacy against cancer cells diminishes proportionally with increasing IC_50_ values. Hela cells exhibited the highest susceptibility to the oil and its nanoemulgel, with IC_50_ values of 60.25 ± 2.4 μg/mL and 25.11 ± 1.3 μg/mL, respectively. B16-F1 cells displayed sensitivity to the oil and its nanoemulgel, evidenced by IC_50_ values of 204.17 ± 1.5 μg/mL and 39.81 ± 0.8 μg/mL, respectively. The impact on Hep-3B cells was noted with IC_50_ values of 7943.28 and 61.65 ± 1.2 μg/mL for the oil and its nanoemulgel, respectively. Conversely, LX-2 cells exhibited the least susceptibility, with IC_50_ values of 7413.1 ± 4.1 μg/mL and 169.82 ± 2.7 μg/mL for the oil and its nanoemulgel, respectively. These determinations were made through drug concentration measurements in the medium. Despite the marginal impact of both *P. granatum* seed oil and its nanoemulgel on cancer cells, the results suggest that the nanoemulgel formulation outperformed the oil counterpart.

### 3.8. Anti-inflammatory activity of *P. granatum* seed oil and its nanoemulgel

**Fig. 7:**
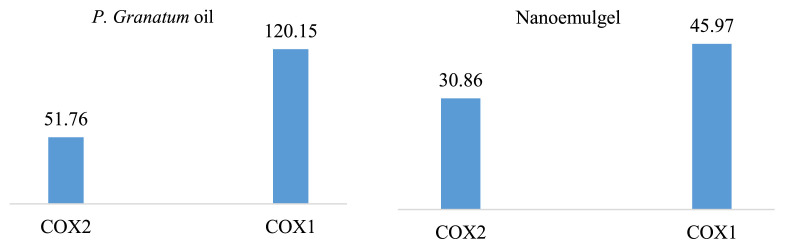
The IC_50_ values (μg/mL) of the anti-inflammatory activity of *P. granatum* seed oil and its nanoemulgel.

The objective of this investigation was to evaluate the anti-inflammatory characteristics of *P. granatum* seed oil and its nanoemulgel. This was accomplished by analyzing their ability to prevent the conversion of prostaglandin H2 from arachidonic acid via cyclooxygenase enzymes (COX-1 and COX-2) at a 50 μg concentration. The findings indicated that the seed oil of *P. granatum* demonstrated inhibitory properties against both COX-1 and COX-2. However, the nanoemulgel composed of *P. granatum* seed oil demonstrated a significant increase in the inhibition of COX-1 (Fig. 7).

The subsequent figures illustrate the IC_50_ values and percentage inhibition of COX-1 and COX2 enzymes by *P. granatum* seed oil and its nanoemulgel. A decrease in the anti-inflammatory effects is observed as the IC_50_ value rises. At concentrations of 50 μg/mL, *P. granatum* seed oil demonstrated inhibition of both COX-1 and COX-2 enzymes. The IC_50_ values for COX-1 and COX-2 were determined to be 120.15 ± 1.3 μg/mL and 51.76 ± 0.5 μg/mL, respectively. On the other hand, the COX-1 enzyme inhibition of the *P. granatum* seed oil nanoemulgel was considerably enhanced, as evidenced by its IC_50_ value of 45.97 ± 0.9 μg/mL. However, no substantial improvement in the inhibition of the COX-2 enzyme was observed.

## 4. Discussion

Since ancient times, essential oils have been known for their medical benefits, so they have been used in many industries, such as perfume, food, beverages, and the pharmaceutical industry. And with the emergence of Islamic civilization, new oil extraction methods began to appear, using new techniques to obtain the full benefit. In the pharmaceutical industry, oils have several roles, including antibacterial, antiviral, anti-diabetic, and anticancer, but due to the differences in the amounts and types of organic compounds, there is a claim that the effectiveness of essential oils is not due to a single mechanism [18].

However, essential oil topical preparations have poor water solubility and bioavailability problems, so the scientists at first made a nanoemulsion preparation to increase solubility, stability, and permeability [19]. However, the challenges of efficiently delivering drugs through the skin's rate-controlling barrier led to the development of nanoemulgels, which incorporate nano-sized oil droplets into a hydrogel matrix for topical drug delivery.

To create a ternary phase diagram, we formulated many mixtures with different ratios of oil, surfactant, and co-surfactant. Following that, we conducted research to evaluate the dimensions of the droplets and the PDI, with the objective of achieving droplet diameters less than 200 nm and a PDI of less than 0.30. The optimal composition in our study comprises Tween 80 and Span 80, with concentrations of 57.6 % and 6.4 %, respectively.

This study used non-ionic surfactants, namely Tween 80 and Span 80, which had hydrophilic-lipophilic balance (HLB) values of 15 and 4.3, respectively. They were selected based on their capacity to produce tiny droplets, little skin irritation, and superior safety in comparison to other surfactants [20]. Additional aspects that have been considered include the kind of emulsifier, its concentration, the ionic strength, the impact of pH, the particle size, and the zeta potential [21,22]. Because of this, Tween 80's high solubility and emulsifying ability helped achieve a low PDI and droplet size.

The nanoemulgel of *P. granatum* seed oil exhibits pseudoplastic rheological behavior, characterized by a decrease in viscosity with increasing shear rate. The optimal formulation achieved a PDI value of 0.214 with an average droplet size of around 190.87 nm. These nanoemulgels were formulated using surfactant, co-surfactant, and different concentrations of Carbopol 940 to optimize spreadability and bioavailability. In addition, the optimal formulation was selected based on criteria such as zeta potential, PDI, droplet size, and rheological properties. The PDI is a crucial parameter for assessing the stability of nanoemulgels, which provide information about the uniformity of particle size distribution. A low PDI suggests that the nanoemulgel exhibits uniformity and a narrow size distribution of particles [16]. Zeta potential provides information on the stability of a dispersion system and the tendency of an emulsion to clump or flocculate by measuring the particle's surface charge or electrostatic potential. The investigation documented a zeta potential measurement of −35 mV, indicating the stability of the system. Hence, this study investigated the production of a nanoemulgel including *P. granatum* seed oil and assessed its antibacterial, anti-inflammatory, and anticancer characteristics.

Carbopol 940 is classified as a swelling or gelling agent due to its ability to increase viscosity and cause swelling [23]. The choice of surfactants for the nanoemulgel formulation is crucial, as they have a significant role in stabilizing the final mixture [19]. They prevent the particles from clumping together by generating strong repulsive forces, which aid in stabilization [24]. Multiple studies have shown that higher surfactant concentrations lead to the formation of smaller droplets. This is because smaller droplets have a bigger surface area. Consequently, a greater amount of surfactant is required to efficiently cover this increased surface area [25,26]. Sungpud et al. (2020) conducted the study [25]. A nanoemulgel containing mangosteen extract was made by mixing Tween 20 and Span 20. This led to the creation of multiple combinations with varying hydrophilic-lipophilic balance (HLB) values, which ranged from 10.2 to 15.1. By employing a combination of surfactants with HLB values of 15.1 and 12.6, they successfully produced nanoparticles with diameters ranging from 18 to 62 nm. Additionally, the zeta potential fell within the range of −39 to −54.5 mV.

Plant oils are mainly used for the treatment of infections produced by bacteria, fungus, or viruses [27]. Infections are widely reported diseases and have been historically treated using plants [28]. This study examined the efficacy of *P. granatum* seed oil and its nanoemulgel in eradicating *Escherichia coli*, *Klebsiella pneumoniae*, *Pseudomonas aeruginosa*, *Proteus vulgaris*, MRSA, *Staphylococcus aureus*, and *Candida albicans*. Kupnik et al. (2021) found that *P. granatum* seed oil had antibacterial properties against *Escherichia coli*, *Klebsiella pneumoniae*, *Pseudomonas aeruginosa*, and *Staphylococcus aureus* [29]. Our investigation found that both the oil and nanoemulgel were ineffective against Proteus vulgaris. However, the *P. granatum* nanoemulgel exhibited more activity than the oil alone. This might be attributed to the tiny size and high surface area of the nanoemulgel particles, which increases the likelihood of their interaction with bacteria and fungus. Another possible explanation is that the presence of oil in nanosized droplets enhanced contact and penetration, resulting in a larger concentration at the desired location [14].

Anwar et al. (2014) found that the antibacterial activity of clove oil nanoemulsion is superior to that of clove oil alone when compared to amikacin antibiotics [30]. Similarly, Sultan et al. (2022) developed a nanoemulgel containing nigella sativa oil and evaluated its antibacterial activity in comparison to the oil alone [31].

Behara et al. (2023) investigated the effectiveness of a nanoemulgel containing turmeric leaf oil in fighting breast cancer cells (MCF-7). The study revealed a notable decrease in cancer cell viability and changes in the structure of cell nuclei. These effects are likely attributed to the enhanced breakdown of hydrophobic substances in turmeric leaf oil, resulting in improved penetration through cancer cell membranes and the initiation of apoptosis [32].

The *P. granatum* seed oil is rich in several chemical compounds, including carbohydrates, steroids, polyphenols, and punicic acid (α-linolenic acid). Notably, punicic acid has diverse anti-cancer activities [33]. We conducted research to assess the effectiveness of *P. granatum* seed oil and its nanoemulgel in fighting cancer. We tested these substances on four different types of cells: LX-2, HeLa, B16-F1, and Hep-3B. The findings of our study revealed that the nanoemulgel formulation of *P. granatum* seed oil exhibited superior anticancer efficacy in comparison to the oil alone, with a notable effect on Hep-3B cells. The reduced particle size (190.87 nm) and greater solubility of the substance allowed for improved penetration and interaction with cancer cells, leading to this outcome.

Astuti et al. (2022) found that a nanoemulgel containing mangosteen rind extract exhibited superior anti-inflammatory activities compared to the gel formulation. The nanoparticles provide an expanded surface area, enhancing the penetration of active substances. This increased penetration through skin layers improves permeability, thereby amplifying the antiinflammatory effects of xanthone molecules [34]. Shukla et al. (2008) discovered that *P. granatum* has the ability to reduce inflammation, particularly by affecting COX-2 [35]. Our research found that *P. granatum* seed oil effectively inhibited the activity of both COX-1 and COX-2 enzymes, with a stronger preference for COX-1. Notably, the nanoemulgel formulation demonstrated comparable effectiveness to the oil against COX-1 but showed significantly enhanced effects against COX-2. The improved solubility and penetration of polyphenols likely contribute to the observed antiinflammatory benefits. The presence of nanoscale oil droplets within the nanoemulgel may be responsible for this enhanced effectiveness.

## 5. Conclusion

To summarize, the current research successfully developed a nanoemulgel containing *P. granatum* seed oil dispersed in water by incorporating the surfactant Carbopol 940 and *P. granatum* seed oil into the nanoemulsion. The *P. granatum* seed oil nanoemulgel demonstrated superior characteristics compared to *P. granatum* seed oil alone, including enhanced stability, reduced droplet size, and increased efficacy against cancer, inflammation, bacteria, and fungi. The optimal formulation was selected based on zeta potential, polydispersity index (PDI), droplet size, spreadability, and rheological properties. *P. granatum* seed oil is a rich source of bioactive compounds, particularly ellagic acid and punicic acid, which exhibit strong antioxidant properties. The *P. granatum* seed oil nanoemulgel shows potential for future applications in developing oral medications with antibacterial, anticancer, and antiinflammatory effects, surpassing the efficacy of pure pomegranate seed oil and those referenced. Additionally, future studies are recommended to focus on investigating the pharmacological effects and safety of *P. granatum* seed oil nanoemulgel in human subjects.

## Figures and Tables

**Table 1:** The selected formulations of *P. granatum* seed oil nanoemulsion.

Tween 80 (%)	Span 80 (%)	Oil (%)	Droplet size (nm)	Polydispersity Index (PDI)
50	15	35	189.44 ± 2.1	0.229 ± 0.09

**Table 2:** Antimicrobial activity of *P. granatum* seed oil nanoemulgel compared with Ampicillin, and Fluconazole antibiotics.

Microorganism	*P. granatum* oil	*P. granatum* oil nanoemulgel	Ampicillin	Fluconazole
*S. aureus* (ATCC 25923)	14 ± 1.5 mm	23 ± 1.7 mm	42 ± 0.7 mm	–
MRSA	18 ± 2.1 mm	29 ± 1.1 mm	26 ± 1.1 mm	–
*E. coli* (ATCC 25922)	17 ± 2.3 mm	28 ± 2.2 mm	33 ± 0.9 mm	–
*P. vulgaris* (ATCC 8427)	Negative (No effect)	Negative (No effect)	38 ± 2.1 mm	–
*K. pneumoniae* (ATCC 13883)	19 ± 1.5 mm	26 ± 1.8 mm	18 ± 1.3 mm	–
*P. aeruginosa* (ATCC 9027)	Negative (No effect)	Negative (No effect)	40 ± 1.2 mm	–
*C. albicans* (ATCC 90028)	12 ± 0.9 mm	18 ± 0.7 mm	–	12 ± 0.1 mm

## Data Availability

The manuscript file contains all of the data that was created or analyzed for this project. I confirm that the study has all the information and consents required.
